# Selection of Effective Moss Control Agents for *Polytrichum commune* and *Marchantia polymorpha* in *Pinus densiflora* Container Seedlings

**DOI:** 10.3390/plants14223417

**Published:** 2025-11-07

**Authors:** Seung-Hyun Han, Ji-Hyeon Lee, Seong-Hyeon Yong, Seon-A Kim, Do-Hyun Kim, Kwan-Been Park, Seung-A Cha, Jenna Jung, Hyun-Seop Kim, Myung-Suk Choi

**Affiliations:** 1 Forest Technology and Management Research Center, National Institute of Forest Science, Pocheon 11186, Republic of Korea; foresthsh@korea.kr (S.-H.H.); khs0607@korea.kr (H.-S.K.); 2 Department of Forest Resources, Gyeongsang National University, Jinju 52828, Republic of Korea; dnlem54@gnu.ac.kr (J.-H.L.); kasiasun06@gnu.ac.kr (S.-A.K.); piment141@gnu.ac.kr (D.-H.K.); qls4347@gnu.ac.kr (K.-B.P.); chai9514@gnu.ac.kr (S.-A.C.); jennajung2002@gnu.ac.kr (J.J.); 3 Division of Forest Biodiversity, Korea National Arboretum, Pocheon 11186, Republic of Korea; ysh1820@korea.kr; 4 Institute of Agriculture of Life Science, Gyeongsang National University, Jinju 52828, Republic of Korea

**Keywords:** terpinyl acetate, moss control, container seedlings, surfactant, phytotoxicity, soil stability, environmentally, friendly herbicide

## Abstract

Moss in container seedling nurseries competes with seedlings for water and nutrients while blocking light, thereby inhibiting growth. This study aimed to address this issue by evaluating the moss control efficacy of 11 chemical compounds, including terpinyl acetate (TA), limonene, and Hinoki essential oil (HEO). The plate experiment results led to the selection of 6 substances (TA, limonene, HEO, pine leaf extract, baking soda, pelargonic acid) that stably controlled both *Polytrichum commune* Hedw. and *Marchantia. Polymorpha* L. When TA, limonene, and HEO were combined with surfactants, moss control rates increased and showed stable performance. In the container seedling experiment, TA, limonene, and HEO demonstrated high moss control effects while exhibiting low growth inhibition. When these three substances were combined with surfactants, the electrolyte leakage index (ELI) decreased, indicating minimal cell membrane damage. Additionally, TA treatment maintained stable soil physicochemical properties with no significant changes in pH or nutrient levels. Microscopic analysis of moss cells showed cell wall deformation and expansion of intercellular spaces in the three substance treatment groups. Future verification of long-term effectiveness, expansion of application targets, and assessment of economic feasibility could lead to the development of eco-friendly moss removal agents for improving container seedling quality.

## 1. Introduction

Bryophytes, comprising approximately 20,000 to 28,000 species, represent the second-largest group of land plants after angiosperms and are taxonomically classified into Anthocerotopsida, Marchantiopsida, and Bryopsida [1,2]. Among these, mosses are non-vascular plants that thrive in humid and shaded environments such as forest floors, wetlands, and nursery surfaces. While ecologically important, mosses become problematic in controlled horticultural and silvicultural systems, especially in containerized seedling production. They form dense mats that hinder seedling emergence, limit water and nutrient uptake, and obstruct light penetration [3,4].

Particularly in forest nurseries, mosses such as *Polytrichum commune* Hedw. and *Marchantia polymorpha* L. proliferate under conditions of high humidity and reduced air circulation, exacerbated by climate change and intensive irrigation regimes. These mosses act as strong competitors, diminishing seedling vigor and complicating nursery management [5]. Moreover, their spread across nursery floors poses operational hazards and fosters continuous re-infestation, necessitating effective and sustainable control methods.

Moss control strategies include the use of chemical herbicides such as flumioxazin, oxyfluorfen, and quinoclamine [3,4,6], light exclusion through shading materials [7], irrigation management to reduce moisture availability [7], and mulching with plant-based materials such as blackcurrant stem pieces or Sphagnum moss [8]. These approaches, tailored to the ecological traits of mosses, offer diverse and increasingly sustainable alternatives for nursery systems.

Chemical herbicides such as flumioxazin, oxyfluorfen, and quinoclamine have been conventionally used for moss suppression in seedling systems [3,4]. However, their repeated application has raised concerns due to phytotoxicity, including leaf chlorosis, inhibited root elongation, and seedling mortality [9]. Beyond plant damage, these chemicals can accumulate in the soil, altering microbial communities, affecting phosphorus and calcium availability, and potentially leading to long-term soil degradation [10,11]. Furthermore, environmental legislation in the EU and North America is increasingly restricting the use of synthetic herbicides in nurseries and urban green spaces, amplifying the demand for safer alternatives.

In this context, plant-derived secondary metabolites, particularly terpenoids, have emerged as promising bioactive compounds for eco-friendly pest and weed management [12]. Essential oils and terpenoid derivatives such as limonene, terpinyl acetate (TA), and hinoki (*Chamaecyparis obtusa* Siebold & Zucc.) essential oil (HEO) exhibit strong antimicrobial and antifungal properties [13,14]. Several studies have demonstrated the potential of essential oils in suppressing pathogens and disrupting biofilms in both agricultural and postharvest settings [15]. Additionally, compounds such as acetic acid, pelargonic acid (PA), and pyroligneous liquor, derived from biomass fermentation or pyrolysis, have shown herbicidal activity [16], while remaining biodegradable and less disruptive to soil ecosystems.

Despite these promising findings, few studies have assessed the application of these natural agents against mosses in forestry nursery conditions. Prior research has either focused on their broad-spectrum herbicidal activity [17,18] or limited their evaluation to in vitro assays without considering field-scale feasibility, phytotoxicity, or soil compatibility. Furthermore, the physicochemical behavior of hydrophobic compounds such as terpenoids on moss surfaces is poorly understood, particularly under conditions where surfactant co-application may enhance their efficacy and absorption [19,20].

This study evaluated the moss control efficacy of 11 compounds, including terpenoid-based natural substances such as TA, limonene, and HEO, against *P. commune* and *M. polymorpha* through both in vitro plate tests and container seedling experiments. The selected moss control agents were further examined in *Pinus densiflora* Siebold & Zucc. container seedlings to assess the enhancement effect of nonionic surfactants, seedling physiological responses, soil property changes, and cellular-level modes of action, aiming to demonstrate the practical potential of low-phytotoxicity, eco-friendly moss control agents.

## 2. Materials and Methods

### 2.1. Moss and Seedling Materials

**Figure 1:**
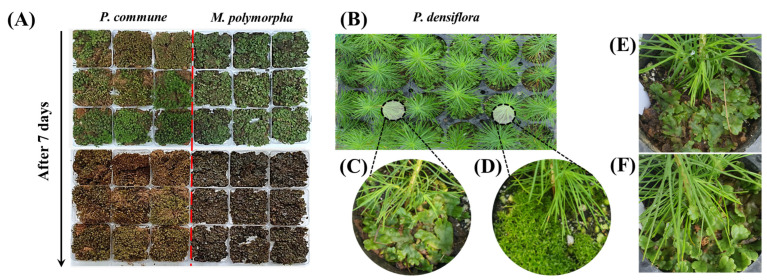
(**A**) *P. commune* (**left**) and *M. polymorpha* (**right**) growing on plates before moss control treatment. The red line in the middle is the dividing line between the two moss species.; (**B**) Moss samples 7 days after treatment, showing clear desiccation and browning of tissues; (**C**) One-year-old *P. densiflora* container seedling used in this study; (**D**) Seedling with established growth of *P. commune*, (**E**) Immediately after *M.polymorpha*, (**F**) 7 days after *M.polymorpha* transplant.

Healthy and vigorous samples of the moss species *P. commune* and *M. polymorpha* were obtained from a commercial moss supplier (Farmer and Beauty, Asan-si, Republic of Korea) (Figure 1A,B), and maintained under controlled greenhouse conditions prior to the experiment. One-year-old container seedlings of *P. densiflora* were grown from seeds collected at a certified seed orchard located in Gangneung, Republic of Korea, and used for the experiment. The seedlings were cultivated in 210 cc plug trays (diameter: 60/45 mm, depth: 93 mm) filled with commercial potting soil (Figure 1C–E). At the start of the experiment, the average height and root collar diameter of the seedlings were 50 cm and 1 cm, respectively. Irrigation was carried out once per day, and no fertilization was applied.

### 2.2. Preparation of Moss Control Agents

A total of eleven moss control agents and one commercial pesticide product were used in this study. These included chemical agents such as baking soda (sodium bicarbonate, CAS No. 144-55-8; Sigma-Aldrich, St. Louis, MO, USA), acetic acid (99.7%; Duksan General Science, Ansan, Republic of Korea), pelargonic acid (CAS No. 112-05-0; Sigma-Aldrich, St. Louis, MO, USA), pyroligneous liquor (TSI, Seongsam, Republic of Korea), ferrous sulfate (FeSO_4_; Junsei Chemical, Tokyo, Japan), zinc sulfate (ZnSO_4_; Junsei Chemical, Tokyo, Japan), α-terpinyl acetate (CAS No. 80-26-2; 2-(4-methylcyclohex-3-en-1-yl)propan-2-yl acetate; Sigma-Aldrich, St. Louis, MO, USA), (+)-limonene (CAS No. 5989-27-5; (4R)-1-methyl-4-prop-1-en-2-ylcyclohexene; Sigma-Aldrich, St. Louis, MO, USA), and Hinoki (*Chamaecyparis obtusa*) essential oil (Hanbeeforest, Jangseong, Republic of Korea).

A commercial herbicide containing 9% quinoclamine (Ikkitan™, Dongbu Hannong, Seoul, Republic of Korea) was used as the reference control, while distilled water served as the negative control.

Additionally, plant extracts were prepared from the leaves of *Quercus glauca* Thunb., *Pinus densiflora* Siebold & Zucc., and *Rumex crispus* L. using the method described in Section 2.3 (Preparation of Plant Leaf Extracts).

The concentration of each substance used in the screening experiment was based on the experimental concentrations reported in previous studies showing sterilization or insecticidal effects. However, since there was no prior information on the moss removal effect, all concentrations from 1 to 30% were tested to determine the effective range.

Among the eleven agents and one pesticide product, solid compounds (e.g., baking soda, FeSO_4_, ZnSO_4_) were dissolved in distilled water based on *w*/*v* ratios, while liquid compounds (e.g., terpinyl acetate, limonene, Hinoki essential oil) were diluted based on *v*/*v* ratios.

Initially, a total of twelve materials (eleven experimental agents and one commercial product) were screened for moss control efficacy. Based on the results of this preliminary screening, six promising agents showing visible desiccation and discoloration effects were selected for further evaluation.

Subsequently, the six selected agents (baking soda, acetic acid, pelargonic acid, pyroligneous liquor, terpinyl acetate, and Hinoki essential oil) and the quinoclamine product were evaluated in field tests (quinoclamine 3% and 5%, baking soda 10%, other substances: 30%).

### 2.3. Preparation of Plant Leaf Extracts

Leaf extracts of *P. densiflora* (PLE), *Q. glauca* (QLE), and *R. crispus* (RLE) were prepared following the general extraction method for plant metabolites [21]. Fresh leaves were dried at 40 °C for 24 h, finely ground, and passed through a 1 mm sieve. Each 15 g of powdered sample was extracted with 100 mL of 70% methanol (*v*/*v*) at room temperature for 24 h under constant stirring. The extracts were filtered through Whatman No. 1 filter paper and concentrated under reduced pressure at 40 °C to obtain the final crude extracts. The extracts were stored in sealed containers at 4 °C in the dark and used within 48 h after preparation.

Seventy percent ethanol was used as the extraction solvent because it can efficiently dissolve both polar and nonpolar compounds in plant extracts [22]. A 24 h extraction period was selected as it provides optimal equilibrium between extraction efficiency and compound stability without causing oxidative or thermal degradation [23].

### 2.4. Application of Moss Control Agents and Evaluation of Treatment Efficacy

Moss samples were transplanted onto 9.2 × 9.2 cm plastic plates and acclimated for one week. Each moss control agent was applied to moss surfaces using a fine brush at a rate of 80 L/a to ensure uniform contact. The moss control efficacy was evaluated visually and photographically 7 days post-treatment using a standardized scoring scale. Moss control rates were calculated with the following formula: moss control rate (%) = controlled area/total area. This experiment was performed on plates in triplicate before application to container seedlings. All treatments were conducted simultaneously under identical controlled environmental conditions in independent plates, which minimized environmental variation among replicates. Therefore, the experiment was performed once, with three independent biological replicates per treatment.

### 2.5. Evaluation of Surfactant Effect

To evaluate the enhancement of control efficacy by surfactants, TA, limonene, and HEO were selected for surfactant combination tests among the candidate agents. These terpenoid-based essential oil components exhibit hydrophobic properties and limited water solubility, suggesting that surfactant addition could enhance dispersion and improve performance. Tween 20 (5%, nonionic; Hanbul Chemical Products Co., Ltd., Seongsam, Republic of Korea) was added to these moss control agents at *v*/*v* ratio. Moss control was then assessed as described above.

### 2.6. Moss Control Trials on Container Seedlings

To assess the practical moss control efficacy of selected agents, *M. polymorpha* and *P. commune* were inoculated onto 1-year-old *P. densiflora* container seedlings cultivated in plug trays. Moss samples were allowed to establish for one week prior to treatment. The 6 selected moss control agents were prepared at appropriate concentrations and applied uniformly to the moss-covered seedling surfaces using a soft brush at a rate of 80 L/a. The control effect was visually evaluated 4 weeks post-treatment. Additional assessments, including seedling physiological condition, growth performance, and soil analysis, were conducted 12 weeks after treatment. Seedling and Soil analysis were conducted on randomly selected seedlings and soils regardless of *P. commune* or *M. polymorpha* treatment groups. Five containers were assigned to each treatment group, and moss control, seedling growth, and phytotoxicity surveys were performed by randomly selecting five seedlings per container.

### 2.7. Evaluation of Phytotoxicity in Seedlings Following Moss Control Treatment

Phytotoxic responses were assessed by visually observing *P. densiflora* container seedlings at 12 weeks after moss control treatment. A five-point damage index was used to quantify the severity of seedling injury, defined as follows: 0 = no visible damage, 1 = slight damage to leaves, 2 = browning of less than one-third of the lower leaves, 3 = browning of more than one-third of the lower leaves, 4 = browning extending to the apical meristem, and 5 = complete necrosis or death. This index enabled a systematic evaluation of phytotoxic effects induced by moss control agents. Phytotoxicity surveys were performed by randomly selecting five seedlings per container.

### 2.8. Seedling Physiological and Growth Responses

Electrolyte leakage index, an indicator of cellular membrane damage, was measured following the method of [24], with slight modifications. 0.05 g of fresh leaf samples were collected from the upper canopy of each seedling and immersed in 10 mL of deionized water. After incubation at 50 °C for 30 min, conductivity was measured (EC1), followed by additional measurement of conductivity after incubation at 121 °C for 15 min (EC2). The electrolyte leakage index was calculated by dividing EC1 by EC2. Conductivity of the solutions was measured using a conductivity meter (VE 4810, Korea Scientifics, Seoul, Republic of Korea).

Seedling growth responses were measured by recording shoot height and root collar diameter before and after treatment using a 30 cm ruler and digital caliper. These measurements enabled evaluation of growth suppression or recovery in response to each moss control treatment. The experiment was conducted with 5 repetitions on 5 seedlings per treatment group.

### 2.9. Analysis of Soil Physicochemical Properties

To evaluate potential changes in soil conditions caused by moss control agents, physicochemical parameters of the potting medium were analyzed 12 weeks after treatment. Soil pH and electrical conductivity (EC) were measured using a pH meter and EC meter, respectively. Available phosphorus (P) content was quantified by spectrophotometry, and exchangeable cations, including potassium (K), calcium (Ca), and magnesium (Mg), were analyzed using inductively coupled plasma spectroscopy (ICP) and atomic absorption spectroscopy. These analyses allowed evaluation of nutrient balance and possible treatment-induced changes in soil fertility.

### 2.10. Microscopic Analysis of Moss Control Mechanism

To investigate the mechanism of action of selected moss control agents, structural changes in *M. polymorpha* cells were analyzed following treatment with TA, limonene, or HEO. Moss tissues were immersed in each treatment solution for 5 min and rinsed three times with distilled water. Samples were stained with 0.5% Congo red for 20 min, washed, sectioned with a scalpel, and observed under a light microscope at 40× magnification. Morphological alterations such as cell wall disruption, increased intercellular space, and organelle damage were recorded. Moss immersed with distilled water served as a negative control.

### 2.11. Statistical Analysis

All experimental data were analyzed using IBM SPSS Statistics 27. One-way analysis of variance (ANOVA) followed by Duncan’s multiple range test was used to identify significant differences among treatment groups (*p* < 0.05). Pearson’s correlation analysis was conducted to assess the relationships among moss control rate, electrolyte leakage, seedling damage index, and growth responses.

To further interpret the differential efficacy of moss control agents, volcano plot analyses were performed using log_2_ fold change and −log_10_ (*p*-value) calculations. This analysis visualized the magnitude and statistical significance of changes in moss control efficacy following surfactant (Tween 20, 5%) addition. Treatments showing significant enhancement (*p* < 0.05, fold change > 0.5) were identified as potential synergistic combinations.

In addition, a hierarchical clustering heatmap was created to compare treatment responses across agents and concentrations. Both visualizations were generated using GraphPad Prism version 10 (GraphPad Software, San Diego, CA, USA), a widely used software for scientific data analysis and publication-quality graphics.

## 3. Results

### 3.1. Effect on Moss Control Agents on Moss Control

**Figure 2:**
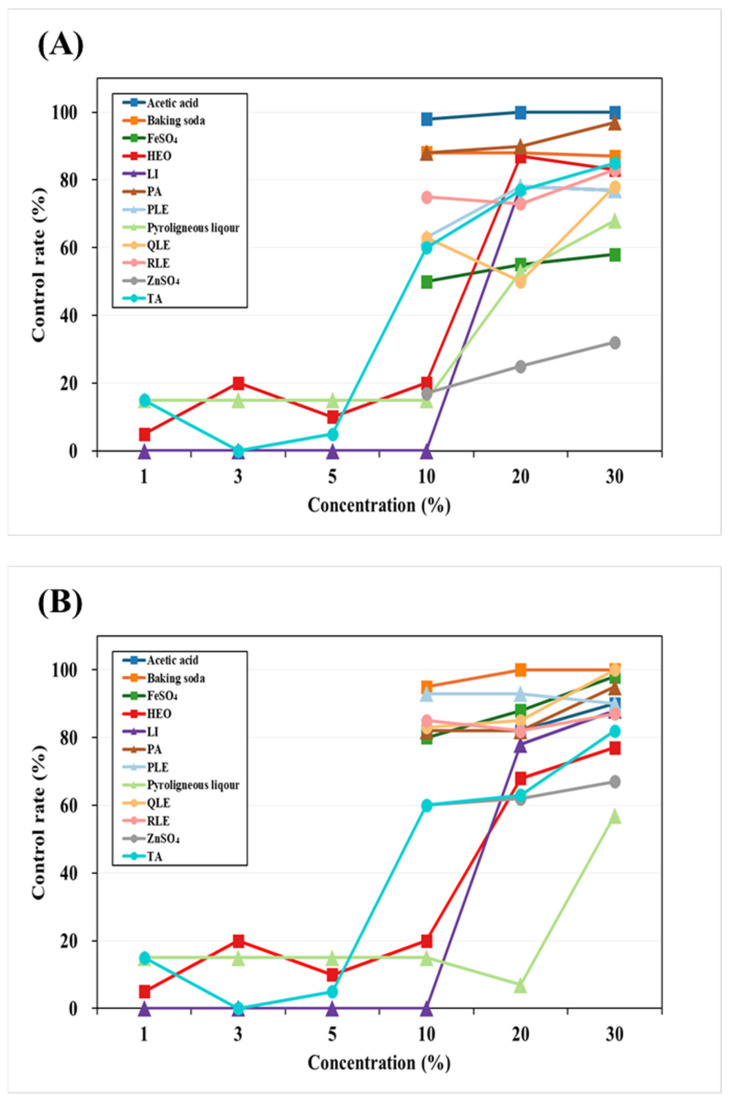
Control by concentration of moss control agent. (**A**) *P. commune*, (**B**) *M. polymorpha*, Li: Limonene, TA: Terpinyl acetate, HEO: Hinoki essential oil, BS: Baking soda, PA: Pelargonic acid, QLE: *Q. gluaca* extract, PLE: *P. densiflora* leaf extract, RLE: *R. crispus* leaf extract. This experiment was performed on plates before application to container seedlings.

This study evaluated the moss control efficacy of 11 chemical and plant-derived agents against *M. polymorpha* and *P. commune* at concentrations ranging from 0% to 30% (Figure 2). Baking soda and PA showed excellent control, achieving more than 80% inhibition at concentrations above 10% against both moss species, suggesting their potential as fast-acting, non-selective herbicides.

Terpenoid-based compounds (TA, limonene, and HEO) also exhibited strong moss control, reaching over 80% efficacy at concentrations above 20%. Notably, TA achieved 63.1% at 20% and 82.1% at 30% against *M. polymorpha*, which was comparable to limonene (83.6%) and HEO (77.1%).

Inorganic salts (ferrous sulfate and zinc sulfate) and pyroligneous liquor were less effective against *P. commune*, showing inconsistent control below 70% even at 30%.

Plant extracts (QLE, PLE, RLE) provided over 80% control against *M. polymorpha* at concentrations above 10%, but their efficacy against *P. commune* was relatively lower.

Overall, moss control efficacy increased in a concentration-dependent manner. *P. commune* exhibited greater resistance compared to *M. polymorpha*. The most pronounced difference was observed with pyroligneous liquor, which showed about a 10% variance at 30% concentration. Based on consistent efficacy across both moss species, TA, limonene, HEO, baking soda, PA, and PLE were selected as candidate agents for further container seedling experiments.

### 3.2. Effect of Surfactant Addition on Moss Control

**Figure 3:**
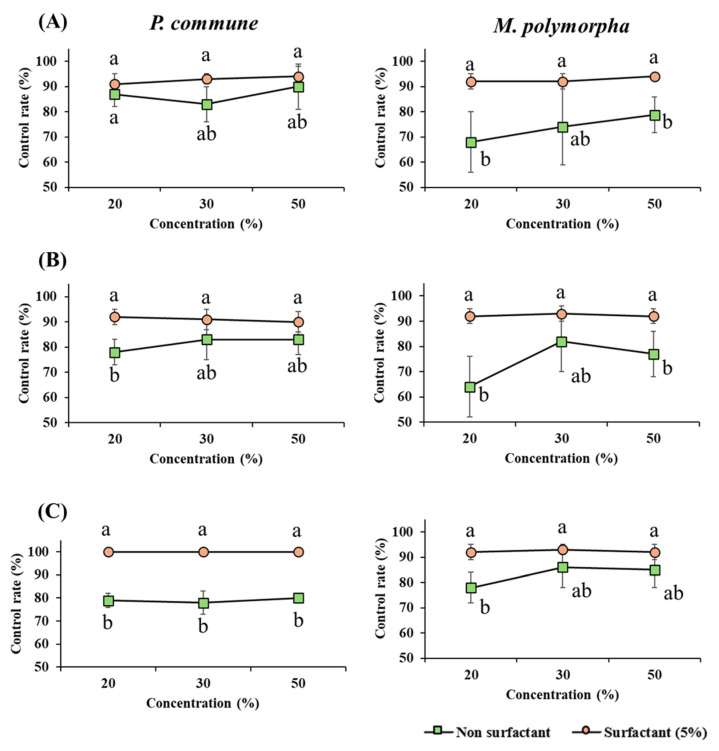
Effect of surfactant mixture treatment on moss control. Moss control efficacy of three terpenoid-based agents with or without surfactant addition. Each graph compares the control rates of *P. commune* (left column) and *M. polymorpha* (right column) at 20%, 30%, and 50% concentrations. The black line (●) represents treatments without surfactant, and the red line (■) indicates treatments with the nonionic surfactant Tween 20 (5%). (**A**) Hinoki essential oil, (**B**) Terpinyl acetate, (**C**) Limonene. Different letters (a,b) indicate statistically significant differences according to Duncan’s multiple range test (*p* < 0.05).

This study examined the effect of surfactant addition on the moss control efficacy of various agents to better understand their delivery efficiency and mechanisms of action. The experimental results showed that the addition of surfactants enhanced moss control in most treatments (Figure 3).

Specifically, HEO at 20% and 30% concentrations against *M. polymorpha* and limonene at 20%, 30%, and 50% concentrations against *P. commune* exhibited control efficacy below 80% before surfactant treatment, but increased to over 90% following the addition of 5% Tween 20. Furthermore, surfactant-treated samples showed reduced variability across replicates, indicating more consistent treatment performance.

Application of the selected moss control agents to *P. densiflora* container seedlings revealed that both *P. commune* and *M. polymorpha* showed no changes in the control (C) and control with surfactant (C + S) treatments. The commercial agent quinoclamine (Q) exhibited control rates of 90% and 97.5% against *P. commune* at 3% and 5% concentrations, respectively, and 87% and 98.75% against *M. polymorpha*.

PA, TA, HEO, and limonene achieved 100% control against both moss species, which was higher than the efficacy of the commercial agent quinoclamine. In contrast, 10% baking soda (BS) and BS with surfactant showed markedly different effects depending on the moss species. Against *P. commune*, high control rates of 96.25% and 95% were observed, whereas against *M. polymorpha* the rates were very low, at 5% and 7%.

**Figure 4:**
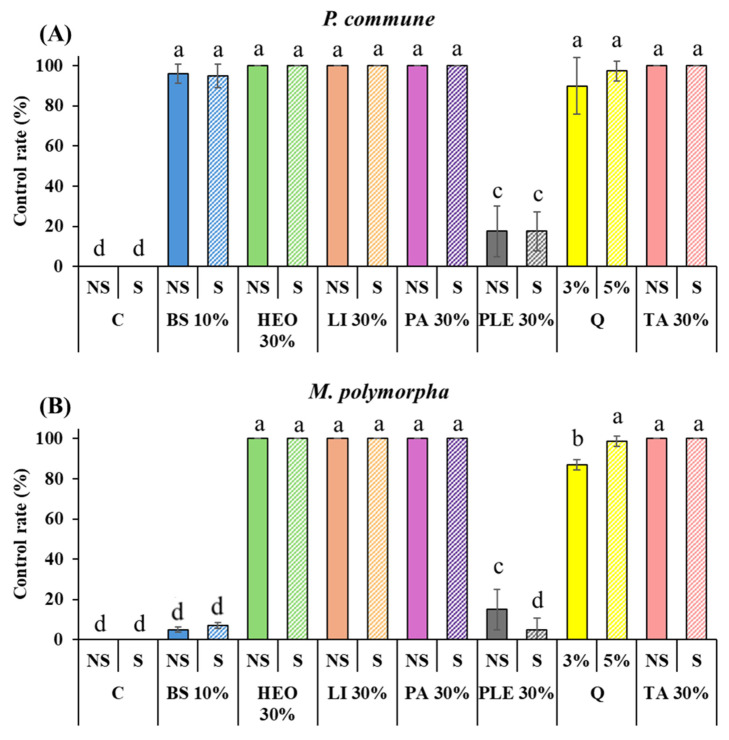
Moss control efficacy of different agents with or without surfactant against *P. commune* and *M. polymorpha*. (**A**): *P. commune* control rate (%), (**B**): *M. polymorpha* control rate, C: Control, C + S: DW + Surfactant 5%, Q: Quinoclamine product, BS: Baking Soda, PA: Pelargonic acid, HEO: Hinoki Essential Oil, TA: Terpinyl Acetate, Li: Limonene, PLE: Pine Leaf Extract. Different letters (a–d) indicate statistically significant differences according to Duncan’s multiple range test (*p* < 0.05).

**Figure 5:**
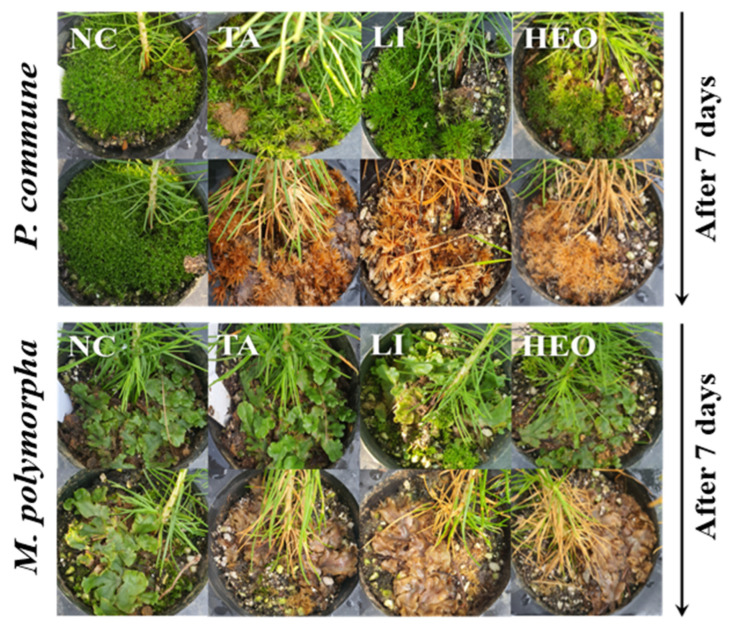
Effectiveness of major moss control agents for controlling *P. commune* and *M. polymorpha* in potted pine seedlings. Representative images showing the effects of three major moss control agents—terpinyl acetate (TA), limonene, and hinoki essential oil (HEO)—on *P. commune* and *M. polymorpha* in potted pine seedlings. Photographs were taken immediately before treatment (0 days) and 7 days after application. TA, limonene, and HEO significantly reduced moss biomass and coverage compared to the untreated control. Visible chlorosis, desiccation, and degradation of moss tissues were observed in treated groups, particularly in TA and limonene treatments.

Similarly, 10% PLE and PLE with surfactant showed poor performance against both moss species, with control rates of 17.5% and 17.5% against *P. commune* and 15% and 5% against *M. polymorpha*. Notably, for *M. polymorpha*, the addition of surfactant further decreased moss control efficacy (Figure 4 and Figure 5).

Figure 4 and Figure 5 illustrate these results. Figure 4 presents quantitative control rates of each treatment against *P. commune* (A) and *M. polymorpha* (B), while Figure 5 shows representative images of TA, limonene, and HEO treatments in *P. densiflora* seedlings, highlighting chlorosis, desiccation, and tissue degradation after 7 days.

### 3.3. Moss Control Effect in P. densiflora Container Seedlings

Application of the selected moss control agents to *P. densiflora* container seedlings revealed that both *P. commune* and *M. polymorpha* showed no changes in the control (C) and control with surfactant (C + S) treatments. The commercial agent quinoclamine (Q) showed control rates of 90% and 97.5% against *P. commune* at 3% and 5% concentrations, respectively, and 87% and 98.75% against *M. polymorpha*. PA, TA, HEO, and Li achieved 100% control against both moss species, which was higher than the commercial agent quinoclamine. 10% baking soda (BS) and that with surfactant showed different control efficacies between the two moss species. Against *P. commune*, high control rates of 96.25% and 95% were observed, while against *M. polymorpha*, the rates were low at 5% and 7%. 10% PLE and that with surfactant showed poor performance against both moss species, with control rates of 17.5% and 17.5% against *P. commune*, and 15% and 5% against *M. polymorpha*. Notably, for *M. polymorpha*, the addition of surfactant resulted in decreased moss control efficacy (Figure 4 and Figure 5).

### 3.4. Physiological Changes and Seedling Damage, Growth of Moss Control Agent-Treated Seedlings

**Figure 6:**
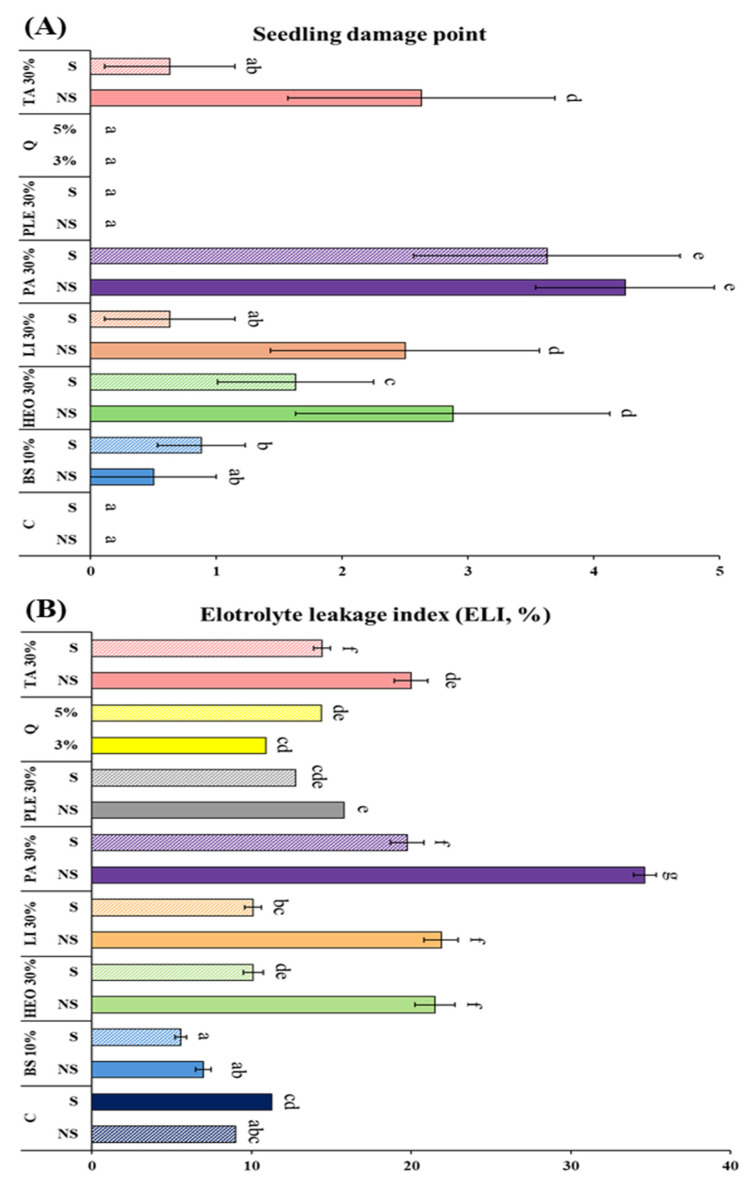
Degree of electrolyte leakage, Seedling damage point and moss control in pine seedlings according to moss control agent treatment. (**A**): Electrolyte leakage index, (**B**): Seedling Damage Point. C: Control, +S: Surfactant 5%, Q: Quinoclamine product, BS: Baking soda, HEO: Hinoki essential oil, Li: Limonene, PA: Pelargonic acid, TA: Terpinyl acetate, PLE: Pine leaf extract. Different letters (a–g) indicate statistically significant differences according to Duncan’s multiple range test (*p* < 0.05).

The physiological changes in *P. densiflora* container seedlings after the application of seven moss control agents were evaluated using the electrolyte leakage index (ELI) and visible seedling damage points (SDP), alongside moss control efficacy (Figure 6). The highest ELI was recorded in 30% PA (34.63%), which also showed the highest SDP (4.25 points), indicating significant phytotoxicity. In contrast, the lowest control rate was observed in 10% baking soda with surfactant (8.2%).

Despite its strong efficacy, PA at 30% caused severe non-selective damage to seedlings, reflecting its known fatty acid-based herbicidal activity. In comparison, terpenoid-based formulations such as TA, limonene, and HEO showed lower ELI and SDP values even at 30% concentration, while maintaining 100% moss control efficacy. When 5% surfactant was added, both SDP and ELI decreased while control efficacy remained at 100%, confirming improved physiological safety. Quinoclamine also exhibited high efficacy (91.88–98.13%) through selective inhibition of photosystem II, while baking soda showed very low ELI (5.56–6.97%) but relatively weak moss control efficacy, particularly against M. polymorpha. Pine leaf extract showed negligible phytotoxicity but remained below 15% in control efficacy.

**Figure 7:**
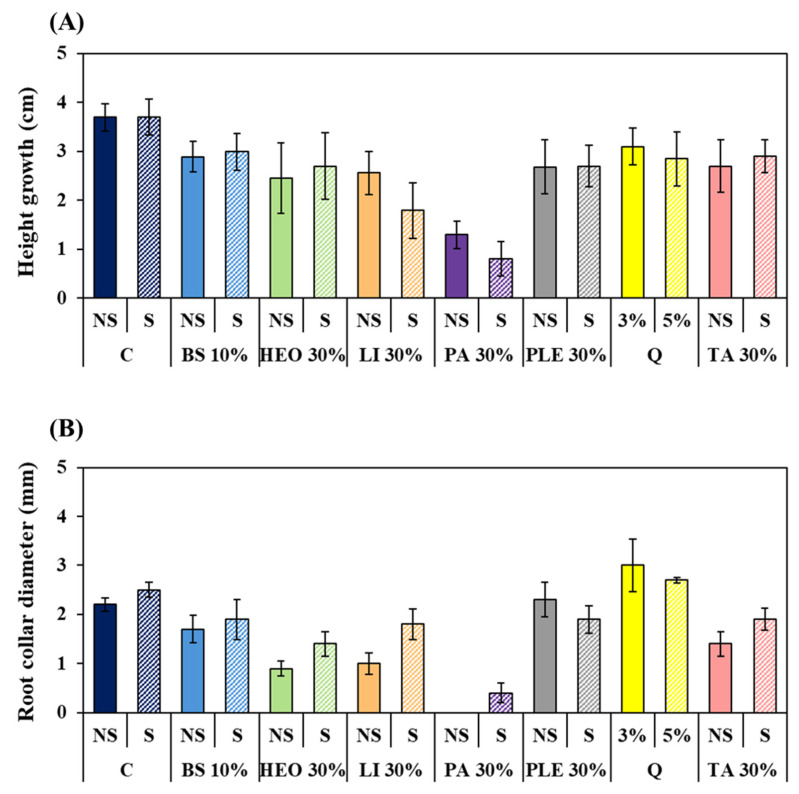
Growth changes in *P. densiflora* seedlings according to moss control agent treatment. (**A**): Height growth change, (**B**): Root collar diameter change. Abbreviation: C: Control, +S: Surfactant 5%, NS: Non Surfactant Q: Quinoclamine product, BS: Baking soda, HEO: Hinoki essential oil, Li: Limonene, PA: Pelargonic acid, TA: Terpinyl acetate, PLE: Pine leaf extract.

Growth responses aligned with physiological indicators (Figure 7). Seedlings treated with 30% PA and PA + S showed the greatest reduction in shoot and root collar diameter growth. In contrast, seedlings treated with TA + S and Li + S showed only slight reductions in shoot growth and maintained stable root collar growth, indicating minimal growth inhibition. HEO + S also reduced inhibition compared to HEO alone, while PLE caused relatively strong growth inhibition. Quinoclamine treatments showed growth nearly identical to the control group.

**Figure 8:**
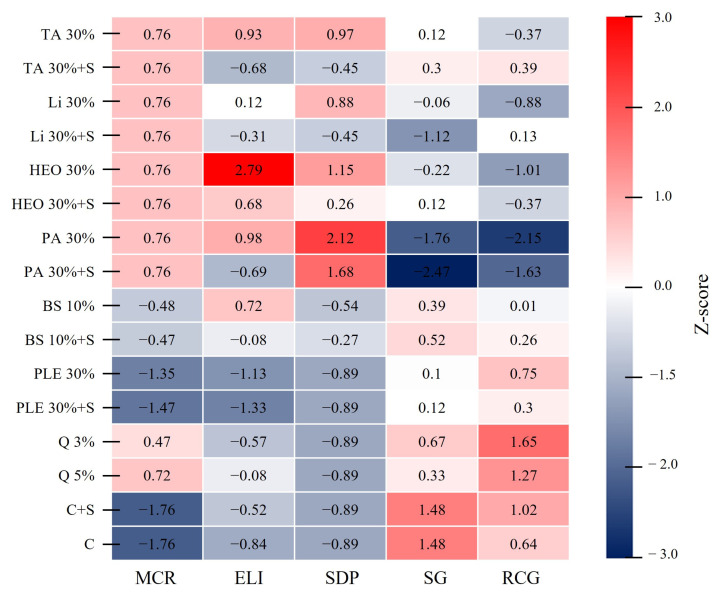
Heatmap analysis of physiological and growth indicators in response to moss control agent treatments. The heatmap presents Z-score normalized values (based on three replicates per treatment) for five key indicators: moss control rate (MCR), electrolyte leakage index (ELI), seedling damage point (SDP), shoot growth (SG), and root collar growth (RCG). Seven treatments were compared: terpinyl acetate (TA), limonene (Li), hinoki essential oil (HEO), pelargonic acid (PA), baking soda (BS), quinoclamine (Q), pine leaf extract (PLE), and control (C). Red cells indicate higher-than-average values, whereas blue cells indicate lower-than-average values. Only statistically significant parameters are shown (*p* < 0.05). Red cells represent higher-than-average values, while blue cells represent lower values.

A heatmap comparing the physiological and growth responses of *P. densiflora* seedlings to each moss control treatment, standardized by Z-scores, is presented (Figure 8). The MCR values were higher than the average for TA, Li, HEO, PA, and Q, whereas BS, PLE, C, and C + S were all lower than the average. The ELI was highest in the 30% HEO treatment (2.79), but decreased to 0.68 when a surfactant was added (HEO 30%+S). Similarly, the addition of a surfactant reduced ELI values in TA, Li, PA, BS, and PLE treatments. The highest SDP value was observed in 30% PA (2.12), while the addition of a surfactant (PA 30%+S) reduced it to 1.68, showing a consistent decrease with surfactant addition. SG was highest in the control (C, C + S) at 1.48, while the lowest value was found in PA 30%+S (−2.47), followed by PA 30% (−1.76). RCG was highest in Q 3% (1.65) and Q 5% (1.27), whereas PA 30% (−2.15) and PA 30%+S showed the lowest values.

Spearman correlation analysis (Table 1) revealed that ELI and SDP were positively correlated with each other and with moss control rate (MCR), while showing negative correlations with shoot growth (SG) and root collar growth (RCG). SDP was strongly negatively correlated with SG and RCG. Although higher moss control rates were expected to improve growth by reducing resource competition, the data showed that higher MCR values coincided with increased seedling damage and reduced growth, suggesting that excessive concentrations of moss control agents caused phytotoxic side effects.

### 3.5. Changes in Soil Physicochemical Properties

Analysis of changes in the physicochemical properties of soil following the application of moss control agents revealed no significant overall alterations, with the TA treatment maintaining the most stable characteristics (Table 2).

Soil pH showed a slight decreasing trend in all treatment groups compared to the control (7.7). The TA, limonene, and HEO treatment maintained relatively stable pH values of 6.9. In contrast, the PA and quinoclamine treatment groups exhibited lower values of 6.8, indicating a shift toward slightly acidic conditions.

Available phosphorus (P) was highest in the pine needle extract treatment group at 9 mg/kg, while the TA and limonene treatments showed levels of 7 mg/kg, similar to the control (8 mg/kg). Quinoclamine treatment groups, however, exhibited the lowest values of 5. This suggests that certain treatments may lead to phosphorus reduction, possibly due to phosphorus fixation or altered microbial activity.

Exchangeable calcium (Ca) levels were generally lower than the control (6.6 cmol+/kg) in most treatments; however, the TA treatment showed an increased value of 7.6 cmol+/kg, suggesting a potential positive effect of this substance on soil cation exchange capacity. Exchangeable potassium (K) and magnesium (Mg) remained stable across all treatments without notable changes.

Electrical conductivity (EC) was consistent at approximately 0.1 dS/m in all treatment groups, except for the pine needle extract group, which showed a slightly elevated value of 0.2 dS/m. This indicates that there was no physiological stress on the soil due to salt accumulation.

### 3.6. Elucidation of the Moss Control Mechanism of Terpenoid-Based Compounds

**Figure 9:**
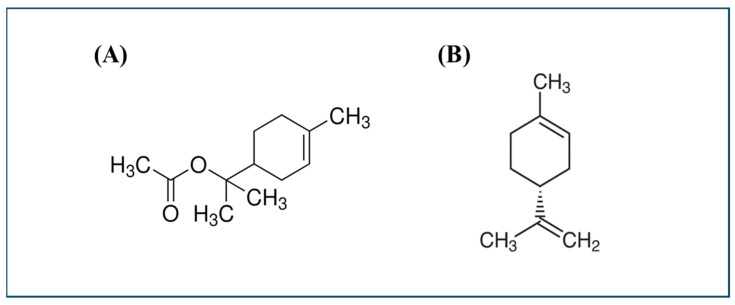
Chemical structures of α-terpinyl acetate (TA, **A**) and (+)-limonene (Li, **B**), the two top-performing terpenoid actives selected for cellular-level mechanism studies.

TA, Li, and hinoki essential oil (HEO) showed strong moss control against *P. commune* and *M. polymorpha* (Figure 9). TA and Li exhibited high control rates with low electrolyte leakage index (ELI), low seedling damage points (SDP), and stable seedling growth. HEO also provided strong control with relatively low phytotoxicity. PA achieved complete control but showed higher ELI and SDP and reduced seedling growth.

**Figure 10:**
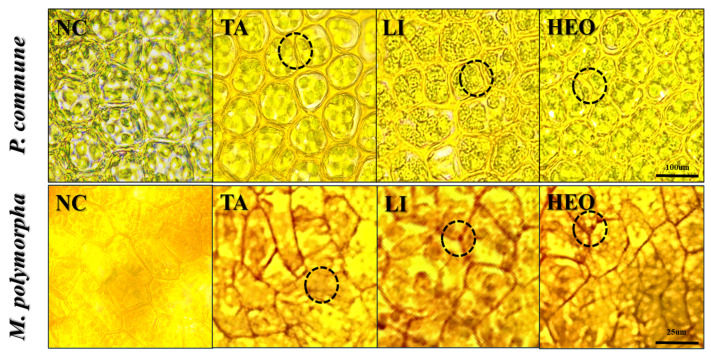
Microscopic observations of *M. polymorpha* cells after treatment with terpenoid-based moss control agents. Representative images showing structural changes in *M. polymorpha* cells following treatment with terpinyl acetate (TA) and hinoki essential oil (HEO). The circle highlights regions where cellular disruption occurred, including alterations in cell wall integrity and intercellular spacing.

To investigate the cellular-level effects of moss control agents, *P. commune* and *M. polymorpha* were treated with TA, limonene, and HEO, stained with 0.5% Congo red, and observed under a light microscope at 40× magnification (Figure 10).

In the negative control groups of both moss species, normal cell morphology was observed, with clearly defined cell walls, uniform staining intensity, and consistent intercellular spacing.

In *P. commune*, TA treatment caused expansion of intercellular spaces due to cell wall damage. Limonene treatment induced swelling of the cell interior and visible damage to the cell wall. HEO treatment showed expansion of intercellular spaces and overall structural disruption.

In *M. polymorpha*, the negative control maintained uniform staining intensity, well-defined cell walls, and consistent intercellular distances, confirming normal structural integrity.

## 4. Discussion

### 4.1. Screening and Evaluation of Effective Moss Control Agents

The evaluation of 11 chemical and plant-derived agents against two representative moss species (*M. polymorpha* and *P. commune*) revealed that baking soda, pelargonic acid, pine leaf extract, and the terpenoid-based compounds (TA, limonene, and HEO) were effective. Baking soda and PA demonstrated rapid inhibition even at low concentrations, confirming their potential as non-selective herbicides, consistent with their mechanisms of cellular dehydration and cuticle disruption [21,22]. Terpenoid-based compounds (TA, limonene, HEO) exhibited strong moss control, likely through mechanisms such as membrane disruption, enzyme inhibition, and oxidative stress. Notably, TA showed high efficacy despite the lack of previous reports on its herbicidal activity, suggesting broader phytotoxic potential [15,23,25]. These findings are consistent with prior reports of limonene inhibiting seed germination and HEO constituents suppressing germination and growth in crop species [13,26,27].

In contrast, inorganic salts and pyroligneous liquor showed low and inconsistent efficacy against *P. commune*, with potential limitations for nursery applications due to risks such as soil acidification [5,10]. Natural extracts (QLE, PLE, RLE) demonstrated promising effects against *M. polymorpha* but were less effective against *P. commune*, indicating the need for optimization and field validation before practical application.

Plant extracts exhibited relatively low moss control activity, likely due to limitations in their chemical composition and mode of action. The 70% methanol extraction used in this study is a standardized method for obtaining various bioactive compounds [21], but it mainly yields water-soluble phenolics and flavonoids with limited cell wall penetration and contact toxicity. The *P. densiflora* extract likely contained terpenes and phenolics, *Q. glauca* tannins and flavonoids, and *R. crispus* anthraquinone derivatives, but these metabolites are typically low in concentration or permeability, resulting in weak efficacy. As most plant extracts contain secondary metabolites such as flavonoids, phenolics, and terpenoids that are less potent than synthetic herbicides [22], further studies should quantify major metabolites to elucidate their mechanisms and practical potential.

### 4.2. The Role of Surfactants in Moss Control

The addition of a surfactant (5% Tween 20) significantly enhanced moss control efficacy across multiple treatments. Notably, control efficacy increased from below 80% to above 90% for 20–30% HEO against *M. polymorpha* and for 20–50% limonene against *P. commune*, while inter-replicate variability decreased, improving treatment consistency. Terpenoid compounds are characterized by low polarity, high volatility, and poor water solubility, which make it difficult to achieve uniform dispersion in aqueous solutions. Therefore, the inclusion of a nonionic surfactant (Tween 20) was necessary to enhance solubility and formulation stability, thereby improving the contact efficiency between the hydrophobic terpenoids and moss surfaces [25,28].

These results are consistent with the notion that surfactants lower surface tension, thereby improving wettability and diffusion and facilitating the surface penetration and delivery of hydrophobic terpenoids [19]. In addition, increased membrane permeability may raise the intracellular delivery of active ingredients, thereby strengthening herbicidal activity [20]. In practical terms, such surfactant-enabled microemulsions improve spreading/retention on thalli and reduce inter-replicate variance by stabilizing droplet size and distribution of hydrophobic terpenoids across the moss surface.

Species-specific anatomical differences also appear to influence the magnitude of the surfactant adjuvant effect. *M. polymorpha*, with its simple thalloid structure, responded more sensitively to terpenoid formulations than *P. commune*, which has multilayered tissues and high water retention. This suggests that surface penetration and on-surface diffusion pathways of the formulations are governed by species-specific tissue architecture [27,29]. Therefore, in field applications, formulation design should optimize the type and concentration of surfactant with consideration of (i) the hydrophobicity/viscosity of the active ingredient, (ii) the tissue and water-retention characteristics of the target moss, and (iii) the wettability of the sprayed surface (substrate).

From an operational standpoint, introducing a surfactant has the potential to achieve target inhibition rates at lower active concentrations or to reduce application volumes, thereby improving both cost-effectiveness and environmental performance. However, excessive increases in membrane permeability may raise the risk of non-target injury (phytotoxicity). Hence, physiological stress indicators such as ELI and SDP should be monitored in parallel, and the active–surfactant combination and dilution ratio should be adjusted stepwise. Furthermore, field-scale verification is required to assess potential long-term impacts on soil and microbial communities under repeated applications.

### 4.3. Moss Control in Container Seedlings

The results obtained from the *P. densiflora* container seedling trials highlight distinct differences in the moss control efficacy and species-specific responses of the tested agents. The commercial agent quinoclamine exhibited high control efficacy against both *P. commune* and *M. polymorpha*, consistent with previous studies demonstrating its selective inhibition of photosystem II electron transport in bryophytes [6]. However, several natural terpenoid-based agents, including PA, TA, HEO, and limonene, achieved complete (100%) moss control, outperforming the commercial standard. This finding reinforces the potential of natural compounds as viable alternatives to synthetic herbicides.

The most striking observation was the differential species response to baking soda (BS). While BS showed high control rates (>95%) against *P. commune*, it was largely ineffective against *M. polymorpha* (≤7%). Such variation suggests that differences in morphology and physiology between the two moss species strongly influence herbicidal efficacy. *M. polymorpha* possesses a thalloid structure with relatively greater resistance to desiccation and chemical stress, whereas *P. commune* exhibits more delicate tissue organization, making it more vulnerable to osmotic and ionic disruption [30]. This species-specific tolerance underscores the necessity of testing potential moss control agents across multiple taxa before broad application.

Pine leaf extract (PLE) was consistently ineffective, even when combined with a surfactant, achieving less than 20% control against both species. This poor efficacy, coupled with negligible phytotoxicity, suggests that not all plant-derived extracts possess the secondary metabolites necessary to disrupt bryophyte physiology. Previous studies have reported that the effectiveness of natural extracts depends heavily on the concentration and type of bioactive terpenoids or phenolics present [31]. Thus, while PLE may be environmentally safe, it lacks the potency required for practical use in nursery settings.

Visual assessments confirmed that TA, limonene, and HEO induced severe chlorosis, desiccation, and structural degradation of moss tissues within seven days of treatment. These symptoms are consistent with the reported ability of terpenoid compounds to disrupt membrane integrity and induce oxidative stress in non-vascular plants [32,33]. The fact that TA, a compound not previously reported for herbicidal activity, demonstrated complete control suggests a broader, previously unrecognized phytotoxic potential. This finding warrants further mechanistic studies to elucidate its mode of action and evaluate its selectivity against vascular plants.

Overall, these results highlight terpenoid-based agents, particularly TA, limonene, and HEO, as promising eco-friendly alternatives for moss control in forestry nurseries. While PA also showed strong efficacy, its non-selective phytotoxicity remains a limitation [31]. Further studies should focus on optimizing application rates and assessing long-term safety to ensure that these natural compounds can replace or supplement synthetic herbicides in sustainable seedling production systems.

### 4.4. Effect of Moss Control Agent on Container Seedlings

The results indicate that while PA, TA, HEO, and Li all achieved complete moss control, their effects on seedling physiology and growth differed significantly. PA was the most phytotoxic, causing the highest ELI, SDP, and severe growth inhibition, which highlights its limitation in nursery environments despite its herbicidal strength. This supports earlier reports that PA is a strong but non-selective herbicide [34].

By contrast, terpenoid-based compounds (TA, limonene, HEO) demonstrated high moss control efficacy while maintaining relatively low phytotoxicity, even at high concentrations. When combined with surfactants, these treatments further reduced physiological stress and growth inhibition, suggesting a synergistic effect that enhances selectivity between mosses and vascular seedlings. This finding aligns with previous research on the selective activity of terpenoids against non-vascular plants [26,31], supporting their potential as eco-friendly bioherbicides for container seedlings.

Baking soda was notable for its low phytotoxicity but insufficient efficacy, especially against *M. polymorpha*. This suggests that while safe, its limited moss control capacity restricts its practical use. Pine leaf extract similarly showed minimal phytotoxicity but very poor efficacy, rendering it unsuitable as a practical agent despite its environmental safety. These results underscore the necessity of balancing efficacy with safety when evaluating moss control agents [31].

Quinoclamine performed consistently, showing high efficacy and minimal immediate effects on seedling growth, consistent with its known selective mechanism [9]. However, long-term inhibitory effects on growth have been documented, indicating that caution is needed in prolonged nursery use.

Correlation analysis emphasized the trade-off between high moss control and seedling health. Positive correlations between MCR and damage indicators (ELI, SDP), coupled with negative correlations between MCR and growth, suggest that current concentrations of moss control agents may be too high. Overuse or misuse of herbicides can lead to yield reductions in crops, and similar patterns appear in moss control for seedlings [35].

Therefore, future studies should focus on optimizing concentrations to achieve moss suppression while minimizing seedling damage. The addition of surfactants and safeners represents a promising strategy, as evidenced by reduced damage and growth inhibition in this study. Tailoring surfactant types and concentrations may further improve performance and provide a safer, more reliable framework for moss control in container seedling production systems [19,20].

### 4.5. Effect of Moss Control Agents on Soil Physicochemical Properties

The analysis of soil physicochemical properties following moss control agent application revealed only minor alterations, suggesting that most treatments did not substantially disrupt soil conditions. This is an important consideration for practical application, as excessive shifts in soil pH or nutrient balance can negatively impact seedling growth and long-term soil health.

Among the agents tested, TA treatment maintained the most stable soil properties, with near-neutral pH (6.9), phosphorus and exchangeable cation levels similar to or slightly improved compared to the control. This stability is consistent with the relatively low phytotoxicity of TA observed in seedling physiological responses, further supporting its suitability for use in sensitive nursery environments. The slight increase in exchangeable Ca (7.6 cmol+/kg) under TA treatment suggests a potential positive effect on soil cation exchange capacity, which may enhance nutrient availability and buffering capacity.

In contrast, PA and quinoclamine treatments induced a shift toward mildly acidic soil conditions (pH 6.8). Acidification following herbicide application has been reported previously [11], often linked to organic acid exudation, microbial activity, or decomposition processes. Although the magnitude of change was modest, prolonged or repeated applications may exacerbate soil acidification, which can reduce nutrient availability and alter microbial community composition.

The reduction in available phosphorus under quinoclamine treatment (5 mg/kg) is also notable. A decrease in available P may result from enhanced fixation or microbial immobilization following chemical inputs [10]. Conversely, pine leaf extract treatment increased available phosphorus (9 mg/kg), although this treatment was not effective as a moss suppressant. Such a response may be associated with the release of organic acids or secondary metabolites from natural products [26].

Other soil parameters, the absence of significant changes in EC (0.1–0.2 dS/m) indicates that none of the treatments caused salt accumulation or osmotic stress, a key factor in maintaining soil quality and preventing damage to seedling roots [35].

Overall, these results suggest that TA and limonene treatments maintain soil stability better than PA and quinoclamine, which induced slight acidification and reduced available phosphorus. From a nursery management perspective, the ability of TA to suppress moss growth while maintaining soil chemical balance is a major advantage for sustainable use. Long-term studies are necessary, however, to confirm whether repeated applications alter soil nutrient dynamics or microbial community structure.

Notably, the physicochemical profile of TA (low polarity, moderate volatility, and ester functionality) likely constrained leaching and sustained only short-term surface residency via partial volatilization, while gradual hydrolysis (to terpineol and acetic acid) limited long-term persistence—together explaining the near-neutral pH and stable nutrient status observed under TA treatment.

### 4.6. Moss Control Efficacy and Phytotoxicity

The results indicate that terpenoid-based natural substances, particularly TA and Li, combine strong moss suppression with low levels of phytotoxicity, making them highly promising candidates for practical moss management. This comparatively low phytotoxicity of TA can be mechanistically rationalized by its hydrophobicity—which restricts systemic uptake into vascular seedlings—and by its ester bond, which undergoes gradual hydrolysis into less toxic, biodegradable products, thereby reducing prolonged cellular stress.

TA is an ester-type monoterpenoid predominantly present in essential oils of *Pimpinella anisum* L. and pine species, widely documented for antimicrobial and antioxidant activities [36]. However, herbicidal or moss control properties have not been previously reported. The present findings suggest, for the first time, that TA may also possess phytotoxic or herbicidal activity. Given its natural origin and relatively low phytotoxicity, TA emerges as an eco-friendly moss control agent suitable for container seedling management and horticultural applications.

HEO, extracted from *Chamaecyparis obtusa*, contains a complex mixture of terpenoids including α-terpinyl acetate. Previous studies have reported its antimicrobial and phytotoxic effects [13,37], such as delayed germination and suppressed shoot elongation in *Brassica rapa* and *Lactuca sativa* [38]. In this study, HEO showed consistent moss control efficacy with relatively low seedling damage. The high TA content likely contributes significantly to its activity, although synergistic interactions with other terpenoid constituents cannot be ruled out.

Limonene also demonstrated strong moss control with acceptable phytotoxicity levels. Although some reduction in seedling growth was observed, it remained within thresholds compatible with container nursery use. Considering its well-established herbicidal properties [17,38], limonene represents another viable candidate for moss management.

PA, while effective in moss suppression, displayed stronger phytotoxicity compared to TA and Li, which may limit its use in sensitive cultivation systems.

Taken together, these findings highlight the potential of terpenoid-based agents—TA, HEO, and limonene—as sustainable moss control solutions. TA and limonene, in particular, provided the best balance between efficacy and seedling safety, while HEO and PA also showed high moss suppression capacity. Further optimization of concentration and formulation is warranted to maximize practical applicability.

### 4.7. Moss Control Mechanism of Terpenoid-Based Compounds

The microscopic analyses suggest that terpenoid-based moss control agents induce selective toxicity by disrupting moss cellular structures through distinct mechanisms.

TA appeared to affect both the cell wall and intracellular organelles, likely by interfering with hydrophobic interactions between cellulose-based walls and lipid bilayers. This disruption reduces membrane stability and weakens intercellular cohesion, leading to visible tissue collapse [39,40].

Limonene likely acted through increased membrane permeability, causing osmotic imbalance, intracellular swelling, and eventual morphological collapse [14].

HEO appeared to weaken intercellular adhesion, thereby reducing tissue-level cohesion and promoting disintegration of moss structures [13].

These distinct disruption patterns align with the previously observed high moss control efficacy and relatively low phytotoxicity of TA and Limonene. Their ability to damage moss tissue while sparing vascular seedlings underscores their selectivity. Moreover, when combined with surfactants, terpenoid-based compounds can overcome hydrophobic surface barriers, enhancing penetration and uniform distribution of active ingredients. This synergy between structural disruption and improved delivery may further enhance overall moss control efficiency [19].

## 5. Conclusions

**Figure 11:**
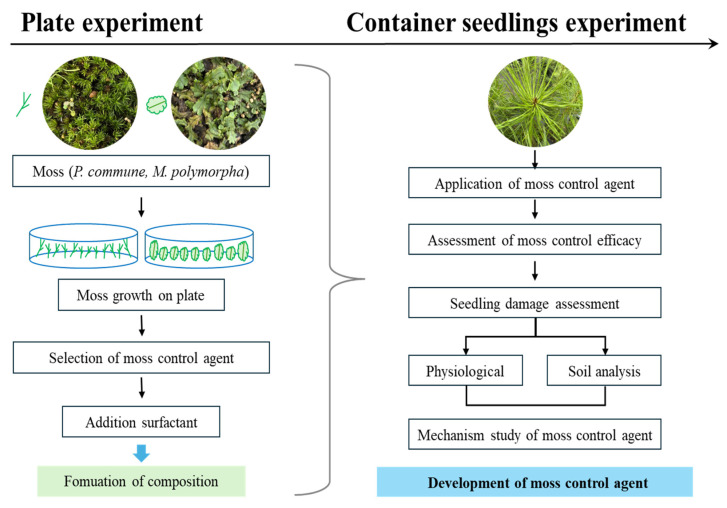
Practical development of moss control agents for nursery applications.

This study established a two-step evaluation system to develop effective and environmentally friendly moss control agents for container-grown *P. densiflora* seedlings (Figure 11). In the initial plate experiment, 11 chemical and plant-derived compounds were tested for their moss suppression efficacy. Several agents including TA, BS, and PA demonstrated high effectiveness, with some achieving control rates of up to 90%. In subsequent container seedling experiments, TA and limonene consistently maintained moss suppression while preserving seedling health and stable soil physicochemical properties such as pH, nutrient availability, and electrical conductivity, indicating low environmental risk. Microscopic observations confirmed that terpenoid components exert their effects by disrupting moss cell structures, such as expanding intercellular spaces and weakening cell walls. This integrated approach—starting with in vitro screening, followed by surfactant combination testing and in vivo validation in seedlings—provides a robust pipeline for developing moss control agents. TA and limonene demonstrated high efficacy, physiological safety, and practical applicability, making them promising alternatives to synthetic herbicides. Future research should focus on long-term effectiveness, safety for non-target organisms, and cost–benefit analyses for large-scale adoption. Ultimately, natural substance-based moss control strategies could offer sustainable and scalable solutions for nursery and forestry applications.

## Figures and Tables

**Table 1:** Correlation analysis between electrolyte leakage, seedling damage, growth parameters, and moss control rate.

	ELI	SDP	SG	RCG	MCR
ELI	1				
SDP	0.743 **	1			
SG	−0.594 *	−0.970 **	1		
RCG	−0.746 *	−0.878 **	0.847 *	1	
MCR	0.525	0.817 **	−0.766 **	−0.695 *	1

Abbreviation: ELI: Electrolyte leakage index, SDP: Seedling damage point, SG: Shoot growth, RCG: Root collar diameter growth, MCR: Moss control rate. *: *p* < 0.05, **: *p* < 0.001.

**Table 2:** Changes in soil physicochemical properties after moss control agent treatment.

Treatment	pH	Organisms (g/kg)	P (mg/kg)	Exchangeable Cation cmol+/kg			EC (dS/m)
				K	Ca	Mg	
Control	7.7	0	8	0.74	6.6	5.1	0.1
Surfactant control (5%)	7.1	0	4	0.67	5.9	4.7	0.1
Baking soda	7.1	0	6	0.66	6.4	4.4	0.1
Pelargonic acid	6.8	0	6	0.67	5.9	3.8	0.1
Terpinyl acetate	6.9	0	7	0.66	7.6	5.1	0.1
Limonene	6.9	0	7	0.56	6.3	4.2	0.1
Hinoki essential oil	6.9	0	7	0.56	6.3	4.2	0.1
pine leaf extract	6.7	0	9	0.56	6.3	4.3	0.2
Quinoclamine product (9%)	6.8	0	5	0.56	6.7	4.5	0.1

## Data Availability

The raw data supporting the conclusions of this article will be made available by the corresponding authors upon request and subject to approval by the National Institute of Forest Science.

## Abbreviations

TA	Terpinyl acetate
HEO	Hinoki essential oil
Li	Limonene
PA	Pelargonic acid
BS	Baking soda
PLE	Pine (*Pinus densiflora*) leaf extract
QLE	*Quercus glauca* leaf extract
RLE	*Rumex crispus* leaf extract
ELI	Electrolyte leakage index
SDP	Seedling damage point
SG	Shoot growth
RCG	Root collar diameter growth
MCR	Moss control rate
EC	Electrical conductivity
ICP	Inductively coupled plasma spectroscopy
NC	Negative control
